# Identifying a Gap in a Cavotricuspid Isthmus Flutter Line Using the Advisor™ HD Grid High-Density Mapping Catheter

**DOI:** 10.19102/icrm.2019.111202

**Published:** 2019-12-15

**Authors:** Daniel R. Frisch

**Affiliations:** ^1^Jefferson Heart Institute, Philadelphia, PA, USA

**Keywords:** Ablation, Advisor™ HD Grid, atrial flutter, electrogram, high-density mapping

## Abstract

This report discusses the mapping of an incomplete cavotricuspid isthmus flutter line with a high-density mapping catheter to visualize the arrhythmogenic substrate responsible for incomplete block. The relevant signals were unapparent when using a traditional ablation catheter but were evident with application of a high-density mapping catheter. High-density mapping holds promise for recording electrograms in gaps in other ablation lesion sets that may not be able to be easily identified using more traditional equipment alone.

## Case presentation

**Figure 1:**
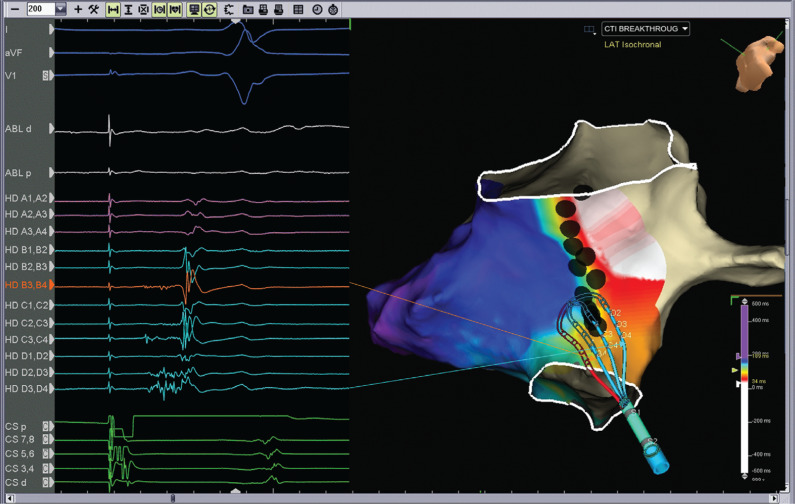
Inferior left anterior oblique views of the right atrium with activation data while pacing from the proximal coronary sinus. The high-density mapping catheter is located proximal to the lesion set where activation across the CTI is observed. Note the high-frequency, low-amplitude, long-duration signals seen on mapping catheter earliest at HD B3,D4 (highlighted in orange) and continuous on HD D3,D4. These identified the target for ablation.

**Figure 2:**
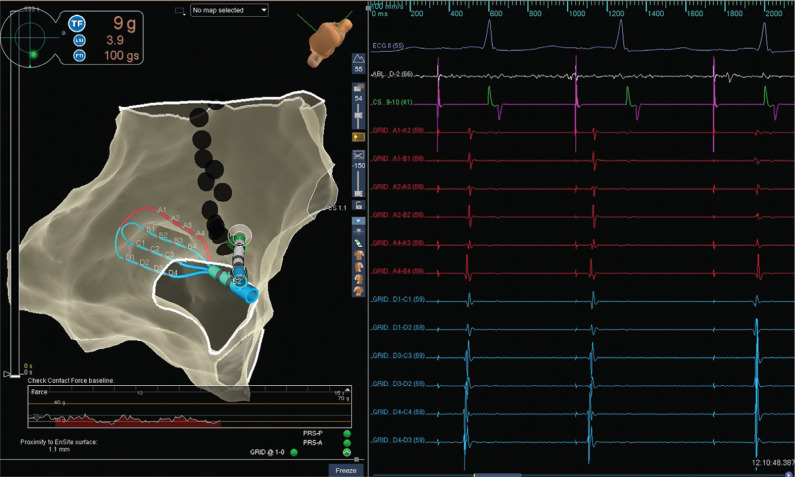
Inferior left anterior oblique view of the CTI with the chosen ablation site for completion of the CTI. A medial-to-lateral block can be observed during ablation at the proximal portion of the CTI. Note that the first beat conducts through the isthmus, that an atrial premature depolarization (APD) is observed during the second beat, and that the third beat demonstrates a medial-to-lateral block in the CTI. A split is seen on the ablation channel once the medial-to-lateral block is observed (arrows). Note that the Advisor™ HD Grid (Abbott Laboratories, Chicago, IL, USA) was moved laterally to avoid interaction with the ablation catheter during ablation.

**Figure 3:**
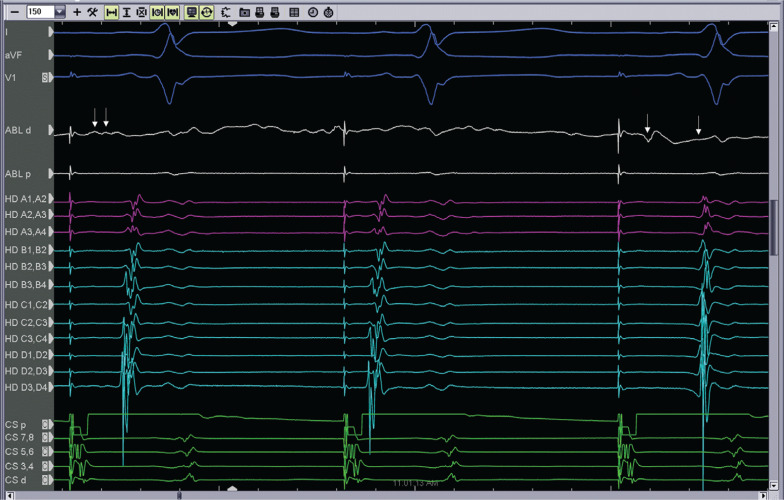
Medial-to-lateral block during ablation at the proximal end of the CTI. Note that the first beat conducts through the isthmus, that an APD is observed during the second beat, and that the third beat demonstrates a medial-to-lateral block in the CTI. The figure also shows splitting of the ablation signal before and after block (arrows).

**Figure 4:**
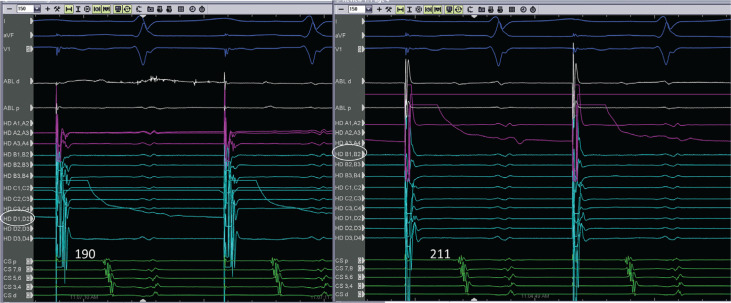
Lateral-to-medial block with differential pacing. When pacing from bipole D1,D2, the isthmus time is 190 ms. When pacing closer to the CTI line on the lateral aspect from bipole B1,B2, the isthmus time is longer at 211 ms.

A 64-year-old male with a history of persistent atrial fibrillation presented for pulmonary vein isolation (PVI). On arrival, his rhythm was a typical atrial flutter. A cavotricuspid isthmus (CTI)–dependent flutter was diagnosed by entrainment maneuvers and by activation mapping using three-dimensional electroanatomic mapping (EAM). During ablation in the CTI, the flutter terminated after the 12th lesion, which was made 10 minutes after the first lesion. However, differential pacing revealed an incomplete lateral-to-medial block. We proceeded to map for the gap in the CTI using a high-density mapping catheter (Advisor™ HD Grid; Abbott Laboratories, Chicago, IL, USA; **Figure 1**). The high-density mapping catheter was placed proximal to the lesion set (near the cavoatrial junction), where activation across the CTI was observed by three-dimensional EAM. High-frequency, low-amplitude, long-duration electrograms were seen on the mapping catheter. These signals were the target for ablation. During ablation at the site of these fractionated electrograms (lesion no. 13), a medial-to-lateral block was observed **(Figure 2)**. One additional lesion was placed to fortify the final site; the total ablation time was 23 minutes. A medial-to-lateral block was also observed using the Prucka EP recording system (GE Healthcare, Princeton, NJ, USA), while differential pacing via the Advisor™ HD Grid (Abbott, St Paul, MN, USA) showed a lateral-to-medial block **(Figures 3 and 4)**. Splitting of the ablation signal was observed as well **(Figure 3)**. Following flutter ablation, we proceeded to map the left atrium with the Advisor™ HD Grid (Abbott Laboratories, Chicago, IL, USA) and perform the PVI procedure.

## Discussion

Ablation for typical atrial flutter is highly successful due in a large part to the bidirectional block achieved across the CTI.^1^ Numerous methods have been reported to determine the completeness of bidirectional block including mapping for double potentials with an isoelectric interval exceeding 110 ms between electrograms along the ablation line (using a 4-mm, electrode-tipped ablation catheter with a 2–5–2-mm interelectrode spacing scheme) or using an incremental pacing technique (with an 8-mm- or 10-mm catheter with dedicated microelectrodes on the catheter tip).^2,3^

In this case, an Advisor™ HD Grid (Abbott Laboratories, Chicago, IL, USA) high-density mapping catheter was employed. This catheter is a 16-electrode, flexible, grid-patterned diagnostic catheter with 3-mm equidistant electrode spacing.^4^ Unlike conventional bipolar catheters, this catheter records in directions both parallel and perpendicular to its recording splines and displays the highest amplitude data on the EAM system. Of note, the high-density mapping catheter was able to record a continuous, low-amplitude, fractionated electrogram **(Figure 1)** that was less apparent when mapping with the ablation catheter (see **Figure 2**, ablation channel). Ablation in the area identified by the high-density mapping catheter quickly led to a medial-to-lateral CTI block. Although empiric ablation might have ultimately worked as well, visualizing the low-voltage isthmus offered a clear target for ablation.

Beyond CTI flutter, this high-density mapping catheter has been used in other ablation scenarios such as mapping for reconnected pulmonary veins during PVI.^5^ This case illustrates the value of high-density mapping to assess the gap during CTI ablation and holds promise for addressing mapping gaps in other lesion sets during the ablation of other arrhythmias.

In conclusion, successful typical atrial flutter ablation requires bidirectional block across the CTI, and a variety of methods are already available to make this assessment. High-density mapping is another tool to use to identify the gap in an incomplete lesion line and direct ablation efforts.
